# Supplementary material for: Development of broad‐spectrum human monoclonal antibodies for rabies post‐exposure prophylaxis
Source: EMBO Mol Med. 2016 Mar 18;8(4):407–21. doi: 10.15252/emmm.201505986 (PMC4818751; doi:10.15252/emmm.201505986)
Supplement: Supplementary file 1 — Appendix [file EMMM-8-407-s001.pdf]

Appendix: Table of Contents

- Appendix Table S1. Selection of blood donors
- Appendix Table S2. Summary of all the genetic and functional characteristics of the panel of 21 isolated human RABV neutralizing antibodies.
- Appendix Table S3. Summary of the 42 RABV isolates tested in this study.
- Appendix Table S4. Summary of the characteristics of the 71 lyssaviruses tested in this study.
- Appendix Table S5. Geographic distribution of the antigenic site I and III polymorphisms of the 2566 RABV isolates analyzed.
- Appendix Figure S1. Neutralization of CVS-11 RABV by RVC20 and RVC58 antibodies combinations.

	CVS-1	ABL	DUV	EBLV-	EBLV-	IRK	KHU	ARA	LB	SHIB	MOK	WCB
Berirab	907	10943	259	138	-	-	143	278	-	-	23	-
RVA	6056	9143	782	2811	130	195	2727	1074	452	76	64	-
RVB	18577	56911	1347	6755	544	212	2543	6209	104	193	448	34
RVC	1486	978	346	1682	136	169	2400	372	97	30	26	32
RVD	3307	751	-	735	77	199	1063	396	-	-	42	-
RVE	2524	18735	507	2391	351	208	2017	953	246	28	159	32
6	1028	3422	621	188	115	38	40	199	-	-	70	-
9	1354	8087	441	630	43	270	66	375	-	-	-	-
10	2235	7463	731	564	325	1022	1220	1045	-	17	697	-
17	511	2155	46	-	-	34	31	116	24	-	45	-
20	4690	30125	94	1411	172	2209	1308	862	18	28	396	-
25	565	23126	75	50	-	661	123	149	-	-	-	-
27	782	27790	110	94	157	541	79	373	-	-	-	-
31	165	5339	-	47	-	-	34	-	-	-	48	-
37	169	258	47	-	-	29	49	272	-	-	71	-
49	178	1120	-	-	-	27	58	102	77	-	-	-
50	2463	3834	145	167	206	625	124	447	-	-	-	-
51	398	2661	56	-	-	35	63	236	-	-	-	-
56	1185	401	147	483	328	225	357	412	-	64	42	-
57	343	3124	64	-	101	212	86	144	-	-	-	-
58	373	365	117	-	38	47	111	173	-	-	32	-
64	867	26957	128	135	29	77	173	162	-	-	43	-
66	62256	10875	171	3606	48565	637	862	1016	-	-	89	-
67	32280	3705	176	6083	10769	194	110	431	-	-	119	-
75	1119	117	68	72	-	57	111	96	-	-	18	-
79	-	-	941	929	419	34880	32	24	-	-	-	-
82	226	26734	62	-	-	40	82	92	-	-	-	-
86	421	1613	78	31	20	24	59	103	-	-	-	-
88	438	1074	29	-	-	-	51	170	93	-	37	-
89	682	3137	78	148	108	94	35	489	-	-	46	-

Appendix Table S1. Selection of blood donors. Neutralization of 12 different pseudotypes lyssaviruses by a panel of 29 plasma samples from RABV vaccinees expresses as ID50 titers.

		Isotype	HEAVY CHAIN						LIGHT CHAIN				CVS-11 pp-neutralization	Neutralization potency ratio vs Berirab	WB	
	Donor		VH	% nts	JH	% nts	DH	HCDR3 lenght	VL/VK	% nts	JL/JK	% nts	(IC90 ng/ml)	folds	Non-red.	Red.
CR057		IgG1 λ	V1-69*01	na	J5*02	na	D3-10*02	na	VL2-11*02	na	JL2*01	na	1	987	++	-
CR4098		IgG1 κ	V3-33*03	na	J4*02	na	D6-19*01	na	VK1-17*01	na	JK1*01	na	8	123	++	-
RAB1		IgG1 κ	V3-33*03	na	J4*02	na	D6-6*01	na	VK3D-11*02	na	JK1*01	na	49	20	ND	ND
RVA122	A	IgG1 λ	V4-59*03	95%	J6*02	89%	D4-17*01	17	VL1-47*01	94%	JL3*02	92%	0,03	32900	+	-
RVA144	A	IgG3 λ	V4-39*01	92%	J5*02	84%	D4-4*01	12	VL1-40*01	97%	JL2*01	92%	0,09	10967	+	-
RVA125	A	IgG1 κ	V2-5*10	96%	J4*02	90%	D6-13*01	15	VK1-12*01	97%	JK4*01	100%	0,5	1974	+	-
RVB185	B	IgG1 λ	V4-39*01	89%	J4*02	85%	D3-9*01	11	VL1-44*01	95%	JL3*02	97%	0,3	3290	++	-
RVB492	B	IgG1 λ	V3-23*01	90%	J4*02	98%	D3-10*01	18	VL-2-11*01	97%	JL2*01	97%	0,2	4935	-	-
RVB143	B	IgG1 λ	V1-46*01	93%	J6*02	90%	D2-21*01	20	VL2-14*01	94%	JL2*01	84%	317	3	++	+
RVB161	B	IgG1 λ	V4-39*07	96%	J4*02	88%	D4-17*01	14	VL1-44*01	98%	JL3*02	100%	0,08	12338	-	-
RVB181	B	IgG3 λ	V3-30*02	91%	J6*02	82%	D1-26*01	15	VL1-40*01	94%	JL2*01	89%	1,4	705	+	-
RVB686	B	IgG1 κ	V3-30*04	95%	J2*01	94%	D6-6*01	15	VK1-5*01	93%	JK1*01	97%	0,6	1645	+	-
RVC03	C	IgG1 κ	V3-23*01	91%	J4*02	94%	D3-3*01	16	VK1-44*01	96%	JK3*02	97%	1	987	+	-
RVC20	C	IgG3 κ	V4-31*06	96%	J4*02	90%	D3-3*01	16	VK1-16*01	98%	JK4*01	100%	0,1	9870	++	-
RVC21	C	IgG3 λ	V4-39*01	93%	J5*01	80%	D4-17*01	13	VL1-51*01	96%	JL1*01	100%	0,04	24675	+	-
RVC38	C	IgG3 κ	V1-69*06	91%	J4*02	85%	D5-24*01	14	VK1-9*01	96%	JK4*04	100%	0,01	98700	-	-
RVC44	C	IgG3 κ	V3-21*01	92%	J3*02	86%	D6-19*01	17	VK1-39*01	95%	JK1*01	100%	0,7	1410	++	+
RVC58	C	IgG1 λ	V3-23*04	96%	J6*02	95%	D2-15*01	17	VL2-14*01	99%	JL2*01	94%	0,01	98700	-	-
RVC68	C	IgG1 κ	V4-59*01	93%	J6*03	89%	D4-17*01	14	VK1-12*01	96%	JK2*01	94%	9	110	+	+
RVC111	C	IgG1 κ	V3-30*04	98%	J4*02	94%	D1-14*01	13	VK1-5*01	96%	JK1*01	95%	0,6	1645	-	-
RVC04	C	IgG3 λ	V3-30*03	95%	J6*02	77%	D1-7*01	15	VL1-40*01	96%	JL3*01	97%	0,02	49350	+	-
RVC56	C	IgG3 κ	V3-7*01	98%	J6*02	98%	D3-10*01	20	VK-1*5*03	98%	JK2*01	97%	0,03	32900	-	-
RVC69	C	IgG3 κ	V1-69*01	92%	J4*02	92%	D5-24*01	16	VK1-9*01	94%	JK4*01	95%	0,13	7592	ND	-
RVD74	D	IgG1 λ	V3-48*02	94%	J4*02	98%	D5-5*01	21	VL2-14*01	92%	JL2*01	91%	0,03	32900	++	-

Appendix Table S2. Summary of all the genetic and functional characteristics of the panel of 21 isolated human RABV neutralizing antibodies. Shown are VH, VL and VK usage, the percentage of nucleotide identity to the corresponding germline gene, the neutralization potency on CVS-11 RABV pseudoviruses expressed as the concentration of IgG in ng/ml able to neutralize 90% of viral infectivity (IC90) and the reactivity of the antibodies in western blot (WB) under non-reducing or reducing conditions.

Isolate name	RVC58	RVC20	CR57	CR4098	RAB1	HRIG (Berirab)	Neutralization assay used*
9704	6	83	63	83	5	188	RFFIT
Botswana/dog/BNVL2009-3580/V5493-2010	2	18	15	6	10	n.e.	FAVN
Botswana/honey badger/BNVL2009-6665/V5493-2010	21	46	7	27	13	n.e.	FAVN
Burkina Faso/dog/139-2007/V6155-2007	46	27	855	3524	908	12192	FAVN
Burkina Faso/dog/9-2007/V6155-2007	0,18	0,42	262	2376	483	12000	RFFIT
02045	1	133	4	445	1	1604	RFFIT
France/red fox/GS7 1-11-1990/V1039-2011	21	46	515	43	3.79	9356	FAVN
Pasteur Virus (PV)-Paris-93127	56	1	56	24	ND	14	RFFIT
9001	63	140	41	130	ND	140	RFFIT
Italy/dog/V3425/2009	3	43	74	33	4.94	12315	FAVN
Italy/human/V117/1996 (Nepal)	1	0,41	55	18	3	7894	RFFIT
Italy/red fox/V673/2011	21	105	378	43	30	n.e.	FAVN
Brazil/bat/260-08/RS1249-2013	415	873	678	60		12192	FAVN
	7,9	6,15	969	11	460	3464	RFFIT
Brazil/bovine/303-2011/RS1040-2012	1,23	9,8	96	31	73	1080	RFFIT
Brazil/kinkajou/RS3609-5-2011	2	2	520	1571	4153	5509	FAVN
Challenge virus strain (CVS)-11	13/16/ 0.1	1171, 20, 0.1	2841	56/440 8/4	4,20	58054/57 78/987	FAVN/RFIIT/PV
9508SAD BERN	7	1	1000	23600	1	221300	RFFIT
Niger/Dog/105-251-2007/V6097-2007	21	27	515	4593	1543	16258	FAVN
04030	6	6	13	543	2	4233	RFFIT
Poland/racoon dog/1985/V3229-2009	20	27	377	33	154	n.e.	FAVN
9141	10	11	22	930	1294	10965	RFFIT
Spain/dog/201020958-2010/RS639-2012	2,23	3,4	121	6,2	3	5288	RFFIT
8743	7	3	66	99	2	1700	RFFIT
Tunisia/dog/ARIANA2-1991/V3951-2010	21	15	113	16	8	n.e.	FAVN
ERA (Evelyn Rokitniki Abelseth)	0.3	0.3	0.8	0.7	<7	1086	PV
CVS 9.13	21	7	24	23600		1700	RFFIT
RV250	21,5	2,2	84,5	0,6	0,2		PV
RV193	12,8	0,6	38,4	0,4	1,4		PV
RV277	13,9	1,5	100,3	0,3	1,3		PV
RV61	15,1	0,08	54,7	0,09	0,07		PV
93033ISR	3,1	2,4	13,5	<0.08	0,1		PV
86081IRA	<7	<7	<7	<7	<7		PV
91004USA	8,7	21	319,5	0,08	8,4		PV
Mauritania/dog/2019-2006/V6235-2007	0,15	0,11	100	359	206	9704	FAVN
Pittman More strain (PM) (PV12?)	66	NA	100	21	NA	11200	RFFIT
87006TU*	+	+	+	+	+		FACS
91001USA*	+	+	+	+	-		FACS
91035OMA*	+	+	+	+	+		FACS
98011CHI*	+	+	+	+	+		FACS
09029NEP*	+	+	-	+	+		FACS
02052AFG*	+	+	+	+	+		FACS
FLRC090148	+/-	+	+/-	+	+		FACS
RV/R3.PHL/2008/TRa-065*	+	+	-	+	+		FACS
CVS11-N336S*	+	+	+	+	+/-		FACS

* tested for binding to G-protein trasfectants

Appendix Table S3. Summary of the 42 RABV isolates tested in this study. Neutralization activity (IC50 for viruses and IC90 for pseudvoviruses in ng/ml) of RVC20, RVC58, CR57, CR4098 and RAB1 monoclonal antibodies and HRIG as illustrated in Figure 4. RFFIT, rapid fluorescent focus inhibition test; FAVN, fluorescent-antibody virus neutralization test; PV, pseudovirus-based neutralization assay. *, viruses tested by FACS for binding to G protein transfectants.

Isolate name	Host species	Country of origin	Year	Viral species	Phylogroup	Virus type	Lineage	Accession number
**9704**	*Tadarida brasiliensis* (Mexican free-tailed bat)	Argentina	1997	RABV	I	V	Bat (American Indigenous)	ABZ81200
**Botswana/dog/BNVL2009-3580/V5493-2010**	*Canis familiaris* (dog)	Botswana	2009	RABV	I	V	Cosmopolitan (ex Africa 1)	KU739040
**Botswana/honey badger/BNVL2009-6665/V5493-2010**	Mellivora capensis (honey badger)	Botswana	2009	RABV	I	V	Africa 3	KU739041
**Burkina Faso/dog/139-2007/V6155-2007**	*Canis familiaris* (dog)	Burkina Faso	2007	RABV	I	V	Africa 2	KU739038
**Burkina Faso/dog/9-2007/V6155-2007**	*Canis familiaris* (dog)	Burkina Faso	2007	RABV	I	V	Africa 2	KU739039
**02045**	*Canis familiaris* (dog)	China	2002	RABV	I	V	Asian 1 or 2a (China)	ABX46649
**France/red fox/GS7 1-11-1990/V1039-2011**	*Vulpes vulpes* (red fox)	France	1990	RABV	I	V	Cosmopolitan-Western Europe	KU739048
**Pasteur Virus (PV)-Paris-93127**	na	France	na	RABV	I	V	na	KU884973
**9001**	Dog (vampire bat virus)	French Guyana	1990	RABV	I	V	Bat (American Indigenous)	ABZ81185
**Italy/dog/V3425/2009**	*Canis familiaris* (dog)	Italy	2009	RABV	I	V	Cosmopolitan-Western Europe	KU739037
**Italy/human/V117/1996 (Nepal)**	*Homo sapiens*	Italy	1996	RABV	I	V	Arctic-like-1	KU739036
**Italy/red fox/V673/2011**	*Red fox*	Italy	2011	RABV	I	V	Cosmopolitan-Western Europe	KC197967
**Brazil/bat/260-08/RS1249-2013**	Bat	Brazil	2008	RABV	I	V	Bat (American indigenous)	KU739047
**Brazil/bovine/303-2011/RS1040-2012**	*Desmodus rotondus* (vampire bat)	Brazil	2011	RABV	I	V	Bat (American indigenous)	KU739045
**Brazil/kinkajou/RS3609-5-2011**	*Potos flavus* (kinkajou)	Brazil	2008	RABV	I	V	Bat (American indigenous)	KU739046
**Challenge virus strain (CVS)-11**	na	Lab strain	na	RABV	I	V/PV	na	EU352767
**9508SAD BERN**	na	Lab strain	na	RABV	I	V	na	ABN11309
**Niger/Dog/105-251-2007/V6097-2007**	*Canis familiaris* (dog)	Niger	2007	RABV	I	V	Africa 2	KU739043
**04030**	*Homo sapiens*	Phillipines	2004	RABV	I	V	Asian 2b (Philippines)	ABX46661
**Poland/raccoon dog/1985/V3229-2009**	*Nyctereutes procyonoides* (raccoon dog)	Poland	1985	RABV	I	V	Cosmopolitan, North-Eastern Europe	KU739049
**9141**	*Vulpes lagopus* (arctic fox)	Siberia, Russia	1988	RABV	I	V	Arctic 2 (Siberia)	KU899138
**Spain/dog/201020958-2010/RS639-2012**	*Canis familiaris (dog)*	Spain (ex Morocco)	2010	RABV	I	V	Cosmopolitan (ex Africa 1)	KU739044
**8743**	*Homo sapiens*	Thailand	1983	RABV	I	V	Asian 2c (south East Asia)	ABX46664
**Tunisia/dog/ARIANA2-1991/V3951-2010**	*Canis familiaris* (dog)	Tunisia	1991	RABV	I	V	Cosmopolitan (ex Africa 1)	KU739050
**ERA (Evelyn Rokitniki Abelseth)**	na	na	na	RABV	I	PV	na	EF206707
**CVS 9.13**	na	Lab strain	na	RABV	I	V	na	O92284
**RV250**	*Urocitellus undulatus* (long-tailed ground squirrel)	Russia (Tuva)	na	RABV	I	PV	Arctic-like	KU534941
**RV193**	*Canis familiaris* (dog)	Pakistan	1989	RABV	I	PV	Arctic-like	KU534940
**RV277**	*Capra aegagrus hircus* (goat)	Pakistan	na	RABV	I	PV	Arctic-like	KU534942
**RV61**	*Canis familiaris* (dog)	India	1987	RABV	I	PV	Arctic-like	KU534939
**93033ISR**	*Canis familiaris* (dog)	Israel	1993	RABV	I	PV	Cosmopolitan, Middle East	KU888637
**86081IRA**	*Canis familiaris* (dog)	Iran	1985	RABV	I	PV	Cosmopolitan, Cosmopolitan, Middle East	KU888638
**91004USA**	*Mephitis mephitis* (skunk)	USA	1991	RABV	I	PV	Arctic related, arctic 1	KU888639
**Mauritania/dog/2019-2006/V6235-2007**	*Canis familiaris* (dog)	Mauritania	2007	RABV	I	V	Africa 2	KU739042
**Pitman Moore strain**	*Canis familiaris* (dog)	Lab strain	na	RABV	I	V	na	AJ871962
**87006TU**	*Vulpes vulpes* (fox)	Turkey	1987	RABV	I	G	Cosmopolitan, America	KU888640
**91001USA**	*Mephitis mephitis* (skunk)	USA	1991	RABV	I	G	Cosmopolitan, America	KU888641
**91035OMA**	Vulpes vulpes (fox)	Oman	1991	RABV	I	G	Arctic related, arctic like 1b	KU888642
**98011CHI**	*Canis familiaris* (dog)	Chile	1998	RABV	I	G	Asia, China, Clade II	KU888643
**09029NEP**	*Bubalus arnee* (wild water buffalo)	Nepal	2003	RABV	I	G	Indian subcontinent	KU888644
**02052AFG**	*Canis familiaris* (dog)	Afghanistan	2002	RABV	I	G	Cosmopolitan, Cosmopolitan, Middle East	KU888645
**FLRC090148**	*Procyon lotor* (raccoon)	USA	2009	RABV	I	G	Cosmopolitan, America	AGE31951
**RV/R3.PHL/2008/TRa-065**	*Canis familiaris* (dog)	Phillipines	2008	RABV	I	G	Asian 2b (Philippines)	BAN14123
**ABLV/Australia/bat/ABL1-1997/V1039-2011**	*Pteropus alecto* (Black flying fox)	Australia	1997	ABLV	I	V		KU739052
**1301 Bokeloh bat lyssavirus**	*Myotis nattererii* (Natterer's bat)	France	2013	BBLV	I	V		KU761304
**86132SA**	*Homo sapiens*	South Africa	1971	DUVV	I	V		KU761302
**DUVV/SouthAfrica/human/1971/RS639-2012**	*Homo sapiens*	South Africa	1971	DUVV	I	V		KU739053
**EBLV1a/France/bat/122938-2002/V3951-2009**	*Eptesicus serotinus* (serotine bat)	France	2002	EBLV-1	I	V/PV		KU739051
**EBLV1b/France/bat/8918-1989**	*Eptesicus serotinus* (serotine bat)	France	1989	EBLV-1	I	V/PV		EU293112
**EBLV2/UK/bat/RV1332-2002/V3951-2009**	*Myotis daubentonii* (Daubenton's bat)	UK	2002	EBLV-2	I	V		GU936871
**94112**	*Myotis dasycneme* (pond bat)	Netheralnds	1989	EBLV-2	I	V		AAX62813
**02053**	*Myotis daubentonii* (Daubenton's bat)	Switzerland	2002	EBLV-2	I	V		KU761301
**Australian bat lyssavirus/RV634**	*Eidolon helvum* (straw-coloured fruit bat)	Australia	1996	ABLV	I	PV		GU936883
**Aravan Virus**	*Myotis blythi (Lesser Mouse-eared Bat)*	Kyrgyzstan	1991	ARAV	I	PV		EF614259
**Duvenhage Virus RSA2006**	*Homo sapiens*	South Africa	2006	DUVV	I	PV		EU623444
**Duvenhage Virus ZIM86-RV131**	*Nycteris thebaica (Egyptian slit-faced bat)*	Zimbawe	1986	DUVV	I	PV		GU936870
**European bat lyssavirus 1.RV20**	*Eptesicus serotinus* (serotine bat)	Denmark	1986	EBLV-1	I	PV		GU936874
**European bat lyssavirus 1.RV9**	*Eptesicus serotinus* (serotine bat)	Germany	1968	EBLV-1	I	PV		EU352768
**European bat lyssavirus 2.RV1787**	*Myotis daubentonii* (Daubenton's bat)	UK	2004	EBLV-2	I	PV		EU352769
**European bat lyssavirus 2.RV628**	*Myotis daubentonii* (Daubenton's bat)	UK	1996	EBLV-2	I	PV		GU936882
**Irkut Virus**	*Murina leucogaster* (greater tube-nosed bat)	Russia	2002	IRKV	I	PV		EF614260
**Khujand Virus**	*Myotis mystacinus* (whiskered bat)	Tajikistan	2001	KHUV	I	PV		EF614261
**MOK**	*Felis catus* (cat)	Zimbabwe	1981	MOK	II	V		NC006429
**Shimoni bat Virus**	*Hipposideros commersoni* (Commerson's leaf-nosed bat)	Kenya	2009	SHIBV	II	PV		NC025365
**West Caucasian bat Virus**	*Miniopterus schreibersii* (Schreibers' bat)	Russia	2002	WCBV	III	PV		EF614258
**8619**	*Eidolon helvum* (straw-coloured fruit bat)	Nigeria	1956	LBV	II	V/PV		KU761303
**Lagos Bat Virus NIG56-RV1**	*Eidolon helvum* (straw-coloured fruit bat)	Nigeria	1956	LBV	II	PV		EF547431
**Lagos Bat Virus SA2004**	*Epomophorus wahlbergi* (Wahlberg's epauletted fruit bat)	South Africa	2004	LBV	II	PV		EF547428
**Mokola Virus NIG68.RV4**	*Crocidura sp.* (shrews)	Nigeria	1968	MOK	II	PV		HM623780
**Mokola Virus 98/071 RA361**	*Felis catus (domestic cat)*	South Africa	1998	MOK	II	PV		GQ500108
**Ikoma virus**	*Civettictis civetta* (African civet)	Tanzania	2009	IKOV	IV	PV		JX193798

Appendix Table S4. Summary of the characteristics of the 71 lyssaviruses tested in this study.

	Ag site I			Ag site III													Number of isolates	RABV deaths/year
	L231	S231	P231	V332	I332	(Others) 332	R333	K333	(Others) 333	N336	D336	S336	(Others) 336	I338	V338	(Others) 338		
**Worldwide**	67,6	17,3	14,7	77	22,9	0,08	96,2	2,77	1,01	90,6	3,59	5,66	0,20	93,7	6,16	0,12	2563	55000
**Africa**	100	0	0	68,8	31,2	0,00	100	0	0,00	40,9	59,1	0	0,00	100	0	0,00	93	24000
**Asia**	92,4	0,09	7,47	65,4	34,5	0,09	99,3	0	0,72	99,4	0,45	0	0,18	99,9	0,09	0,00	1111	31000
**Europa**	95,8	0,42	0,42	99,6	0,42	0,00	100	0	0,00	100	0	0	0,00	93,7	6,33	0,00	237	<10
**North America**	21,9	47	31,1	88	11,9	0,11	91,6	7,37	0,99	80,3	3,41	16	0,33	85	14,6	0,33	909	<10
**South America**	87,8	7,04	5,16	69	31	0,00	93,9	1,88	4,23	99,5	0,47	0	0,00	95,8	4,23	0,00	213	<10

Appendix Table S5. Geographic distribution of the antigenic site I and III polymorphisms of the 2566 RABV isolates analyzed.

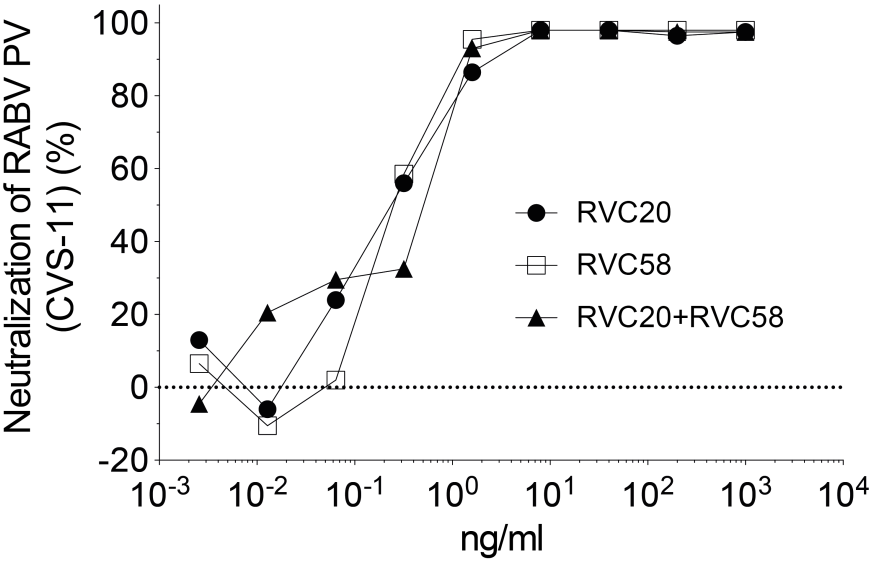

	IC50	IC90
RVC20	0.2275	2.237
RVC58	0.2651	0.784
RVC20+RVC58	0.5451	1.447

Appendix Figure S1. Neutralization of CVS-11 RABV by RVC20 and RVC58 antibodies combinations. RVC20 and RVC58 antibodies were tested individually or as 1:1 cocktail. Shown is the concentration of total amount of the two antibodies in the cocktail.
