# Supplementary material for: Development of broad‐spectrum human monoclonal antibodies for rabies post‐exposure prophylaxis
Source: EMBO Mol Med. 2016 Mar 18;8(4):407–21. doi: 10.15252/emmm.201505986 (PMC4818751; doi:10.15252/emmm.201505986)
Supplement: Supplementary file 2 — Expanded View Figures PDF [file EMMM-8-407-s002.pdf]

Expanded View Figures

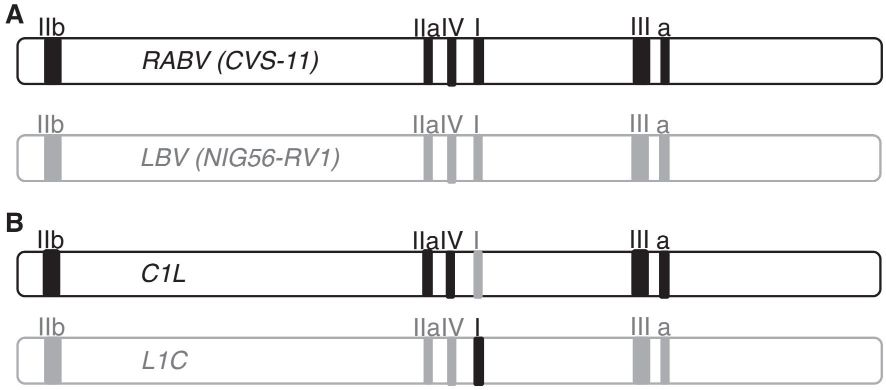

Figure EV1. Schematic showing generation of epitope swapped G proteins.

A A Wild-type G genes from RABV (CVS-11) and LBV (NIG56-RV1) isolates are shown with known epitopes highlighted by the solid boxes. Using overlap extension PCR (Heckman & Pease, 2007), we were able to generate matching sets of G genes for which each epitope in turn had been swapped from CVS-11 to LBV and vice versa.

B Examples of the chimeric G genes generated when antigenic site I was swapped.

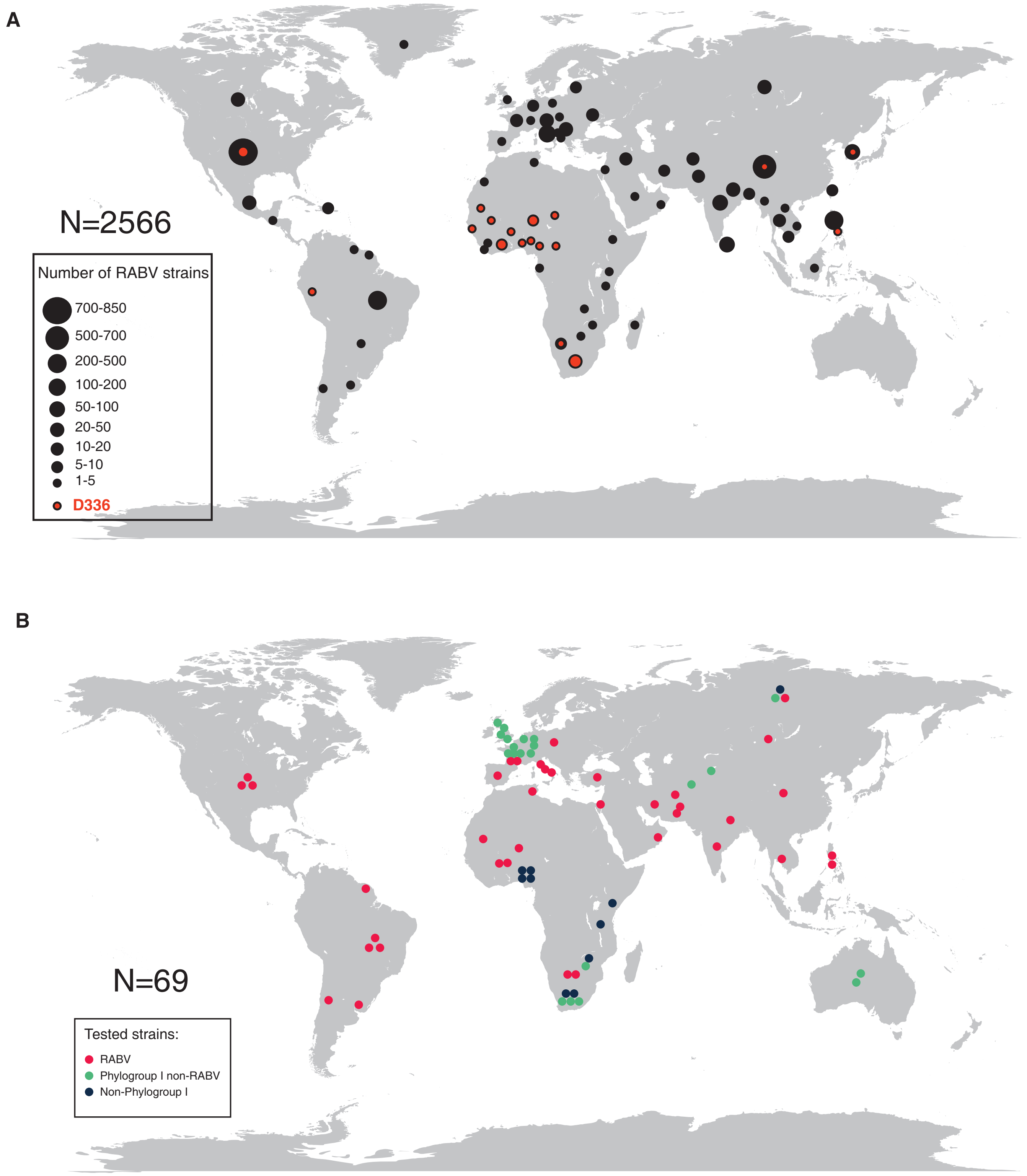

Figure EV2. World maps showing the geographic distribution of the analyzed lyssaviruses.

A Geographic origin of the 2,566 RABV strains used for the analysis of RVC58 and RVC20 epitope conservation. Dot size is proportional to the number of isolates as indicated in the legend.

B Geographical origin of the viruses tested in this study.

 © 2016 Humabs BioMed SA

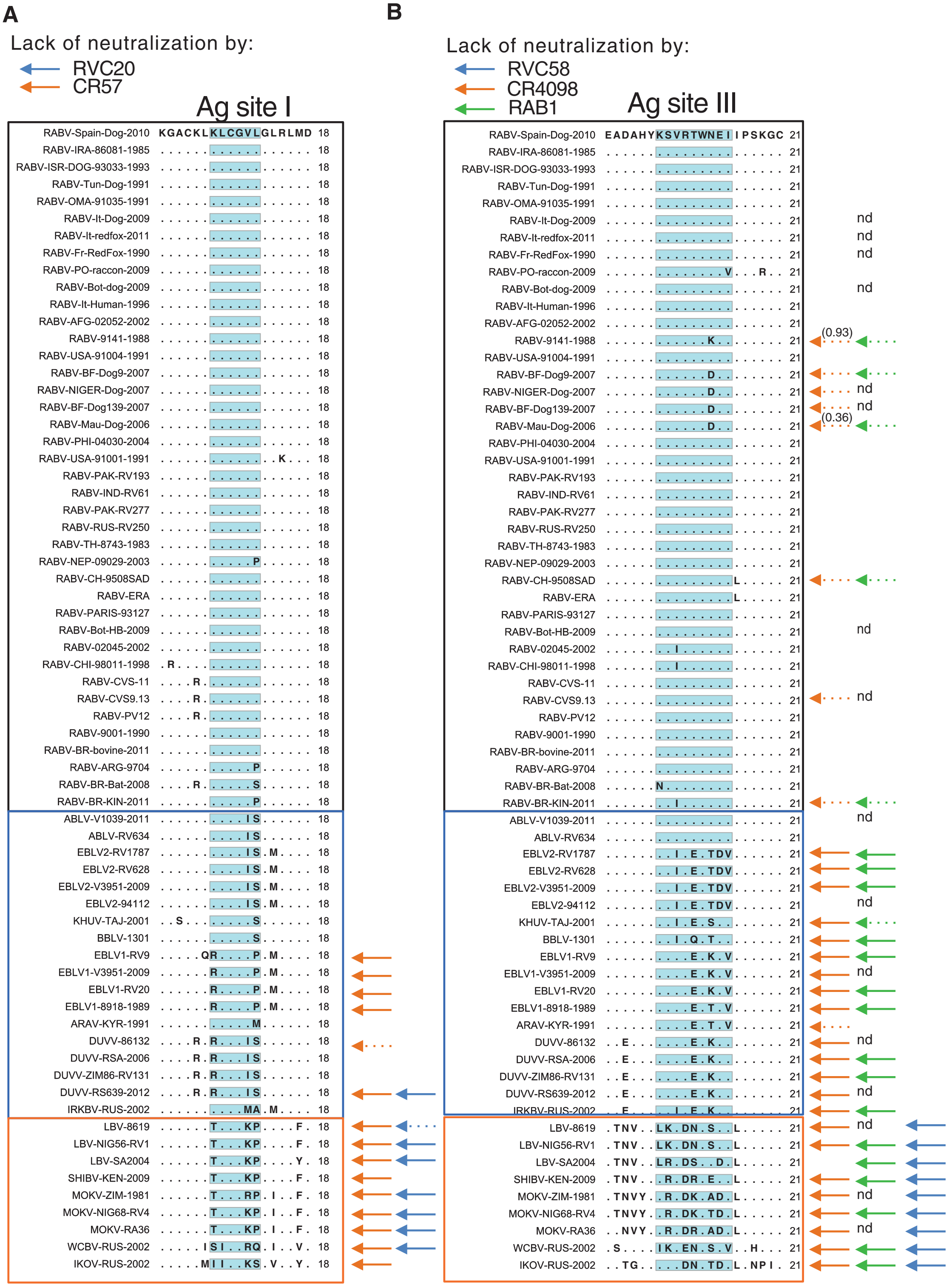

Figure EV3. Alignment of antigenic site sequences of the G protein from all the tested lyssaviruses.
Highlighted in blue and orange are phylogroup I non-RABV and phylogroup II-IV non-RABV lyssaviruses, respectively. Nd, not tested.
A Antigenic site I.
B Antigenic site III.

A

Position	Consensus sequence KLCGVL	Amino acid Frequency (%)	Neutralization by RVC20	Neutralization by CR57
226	K.....	99,73	+	+
	R.....	0,2	+	-
231	L	67,65	+	+
	.....S	17,30	+	+
	.....P	14,73	+	+
	.....H	0,31	?	?

B

Position	Consensus sequence KSVRTWNEI	Amino acid Frequency (%)	Neutralization by RVC58	Neutralization by CR4098	Neutralization by RAB1
330	K........	99.61	+	+	+
	N........	0.27	+	+	+
331	.S.......	99.96	+	+	+
332	..V......	77.05	+	+	+
	..I......	22.88	+	+	+
333	...R.....	96.22	+	+	+
334	....T....	99.65	+	+	+
	....N....	0.35	-*	-*	-*
335	.....W...	99.73	+	+	+
	.....L...	0.27	?	?	?
336	......N..	90.57	+	+	+
	......D..	3.59	+	-	-
	......S..	5.65	+**	-	-
	......G..	0.12	?	?	?
	......K..	0.08	+	-	-
337	.......E.	99.61	+	+	+
	.......D.	0.35	+	-	-
	.......K.	0.04	?	?	?
338	I	93.73	+	+	+
	V	6.16	+	+/-	+/-

*, phylogroup 2 viruses carry N in this position and are not neutralized by either RVC58 and CR4098
**, phylogroup 2 viruses carry S (LBV) and not neutralized by RVC58
+/-, indicates that the Ab neutralizes some but not all viruses carrying the indicated residue

Figure EV4. Level of amino acid residue conservation in antigenic site I (A) and antigenic site III (B) calculated by the analysis of the G-protein sequences from 2,566 RABV isolates as shown in Fig 6.
A, B Shown is a summary of the viruses neutralized by the indicated monoclonal antibodies.

 © 2016 Humabs BioMed SA
